# Neuroanatomical correlates of the perception of body axis orientation during body tilt: a voxel-based morphometry study

**DOI:** 10.1038/s41598-021-93961-8

**Published:** 2021-07-19

**Authors:** Keisuke Tani, Satoshi Tanaka

**Affiliations:** 1 Laboratory of Psychology, Hamamatsu University School of Medicine, 1-20-1 Handayama, Higashi-ku, Hamamatsu, Shizuoka 431-3192 Japan; 2 Faculty of Psychology, Otemon Gakuin University, 2-1-15 Nishi-Ai , Ibaraki, Osaka 567-8502 Japan

**Keywords:** Perception, Human behaviour, Sensory processing

## Abstract

Accurate perception of the orientations of the body axis and gravity is essential for actions. The ability to perceive these orientations during head and body tilt varies across individuals, and its underlying neural basis is unknown. To address this, we investigated the association between inter-individual differences in local gray matter (GM) volume and inter-individual differences in the ability to estimate the directions of body longitudinal axis or gravity during whole-body tilt using voxel-based morphometry (VBM) analysis in 50 healthy adults (20–46 years, 25 men and 25 women). Although no anatomical regions were identified relating to performance requiring estimates of gravitational direction, we found a significant correlation between the GM volume in the right middle occipital gyrus and the ability to estimate the body axis orientation. This finding provides the first evidence on neuroanatomical substrates of the perception of body axis orientation during body tilt.

## Introduction

Accurate awareness of the orientations of the body axis and gravity is important for action. The former as an egocentric (body-centered) reference frame and the latter as an allocentric (gravity- or earth-centered) reference frame together serve as a basis for localizing and orienting external objects in space. Perception of these directions may be related to performance in goal-oriented behaviors and postural control^1–3^.

The central nervous system (CNS) models the body axis and gravity directions by integrating various types of sensory information such as visual, somatosensory, and vestibular signals^4–8^. Previous studies have explored which cortical regions mediate multisensory integration processing. Functional magnetic resonance imaging (fMRI) studies have shown bilateral activation in areas of the occipito-temporal region^9, 10^ and parieto-frontal network such as the posterior parietal and superior frontal cortex^10–12^ when participants judged the location of a visual target relative to the *body axis* (i.e., body-centered judgment). Electroencephalograph (EEG) and transcranial magnetic stimulation (TMS) studies have shown the involvement of bilateral occipito-temporal and left parieto-occipital regions^13^ and the right temporoparietal junction (rTPJ) in judgments of the orientation of a visual line relative to *gravity* (i.e., gravity-centered judgment)^14, 15^.

Although these studies suggest common and distinct neural substrates for the perception of the orientations of body axis and gravity, methodological concerns may affect their validity. First, in the fMRI studies^9–12^, because the display’s edges were not completely hidden, the participants might localize the target concerning the edge (i.e., allocentric reference frame) rather than the body axis, even during the body-centered judgment. Second, in the EEG^13^ and TMS studies^14, 15^, participants judged the visual line orientation in a seated position in which the participant’s body (trunk) and gravity were spatially aligned, and thus, it is unclear whether they judged the orientation of the line relative to body axis or gravity. These concerns raise questions about whether these cortical regions are involved in the perception of the body axis and/or gravity orientations.

Another possible approach to investigate the neural basis underlying the perception of the orientations of body axis and gravity is voxel-based morphometry (VBM). VBM is a neuroimaging analysis involving voxel-wise comparison of local concentrations of gray matter (GM) based on structural brain images^16, 17^. Because the measurements of brain image and behavioral performance are temporally segregated, the VBM approach does not restrict the participants’ posture or the method of stimulus presentation during behavioral measurement. This enables assessment of perceived orientation of body axis and gravity independently.

Thus far, two behavioral tasks have been largely used to evaluate the perception of orientations of body axis and gravity: the subjective visual body axis (SVBA) and subjective visual vertical (SVV) tasks. Participants are asked to align a visual line along the body longitudinal axis for the SVBA task^2, 18, 19^ or along the gravitational direction for the SVV task^1, 3, 7^. Although both tasks are performed accurately in an upright position, SVBA and SVV deviate from the actual direction when the body is laterally tilted^18–21^. However, the extent of these biases largely varies across individuals^2, 22^. Therefore, the primary aim of the present VBM study was to determine the cortical regions where GM volume is correlated with individual differences in the bias of SVBA or SVV caused by whole-body tilt.

The secondary aim of the present study was to assess the correlation between the GM volumes and the extent of individual reliance on visual cues, referred as to “visual dependency”, when estimating the directions of body axis or gravity. In addition to body orientation in space, visual background cues influence the perceived directions of the body axis^23^ and gravity^24, 25^. Although a previous study has shown the involvement of the left posterior parietal cortex (PPC) in visual dependency when estimating the visual vertical^26^, no explorative study has investigated the neuroanatomical correlates of visual dependency at the whole brain level. In this study, we explored the cortical regions where GM volumes correlated with the inter-individual differences in visual dependency for the perception of the directions of the body axis or gravity.

## Results

### Behavioral results

**Figure 1:**
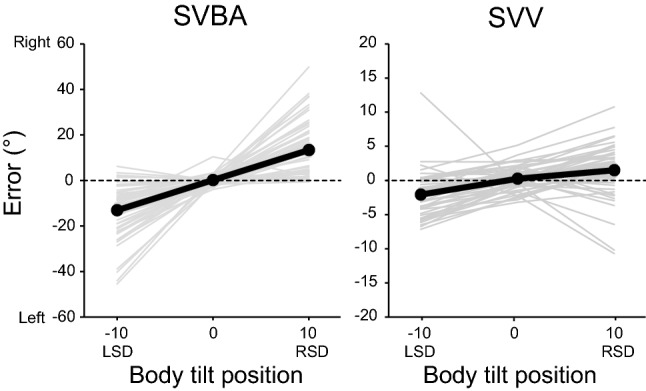
Adjustment errors in the No-OKS conditions at each tilt position for the subjective visual body axis (SVBA) and subjective visual vertical (SVV) tasks. Gray-colored lines represent the averaged error of each participant, and black-colored lines represent errors averaged across all participants. *LSD* left-side-down, *RSD* right-side-down.

Figure 1 shows the individual and group mean errors in the SVBA and SVV tasks in the absence of visual background motion (no optokinetic stimulation (No-OKS) condition) at upright (0°), leftward (left-side-down; LSD) or rightward (right-side-down; RSD) tilted positions. Positive and negative values correspond to rightward and leftward deviations, respectively. In the upright position, group mean errors in the SVBA and SVV tasks were close to zero (mean ± standard errors; SVBA, 0.14° ± 0.31°; SVV, 0.25° ± 0.24°). At tilted positions, errors in the SVBA and SVV tasks were biased toward the direction of body tilt, although the magnitude of the bias was larger for the SVBA (LSD, − 13.05° ± 1.75°; RSD, 13.34° ± 1.77°) than for the SVV (LSD, − 2.07° ± 0.47°; RSD, 1.50° ± 0.56°).

**Figure 2:**
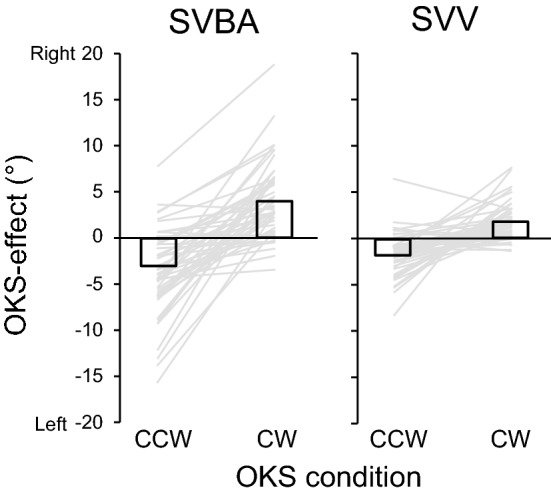
Effect of optokinetic stimulation (OKS-effect) on adjustment errors in the SVBA or SVV tasks. Gray-colored lines represent averaged values of each participant, and black-colored bars represent errors averaged across all participants. As illustrated, both SVBA and SVV were biased toward the rotation direction of OKS. *CW* clockwise, *CCW* counterclockwise.

Figure 2 shows the effects of the OKS (OKS-effect) on the performance of each orientation task. The OKS-effect of clockwise OKS (CW-OKS) was − 3.74° ± 0.66° for the SVBA task and − 1.78° ± 0.33° for the SVV task, and that of counterclockwise OKS (CCW-OKS) was 4.13° ± 0.59° for the SVBA task and 1.80° ± 0.29° for the SVV task, which indicates that perceived directions of body axis and gravity were biased rightward by CW-OKS and leftward by CCW-OKS. As with the effect of body tilt, the amount of OKS-effect tended to be larger for SVBA than for SVV. These results indicate that body tilt and visual background motion influenced the accuracy in estimating the directions of body axis and gravity.

### VBM results

**Figure 3:**
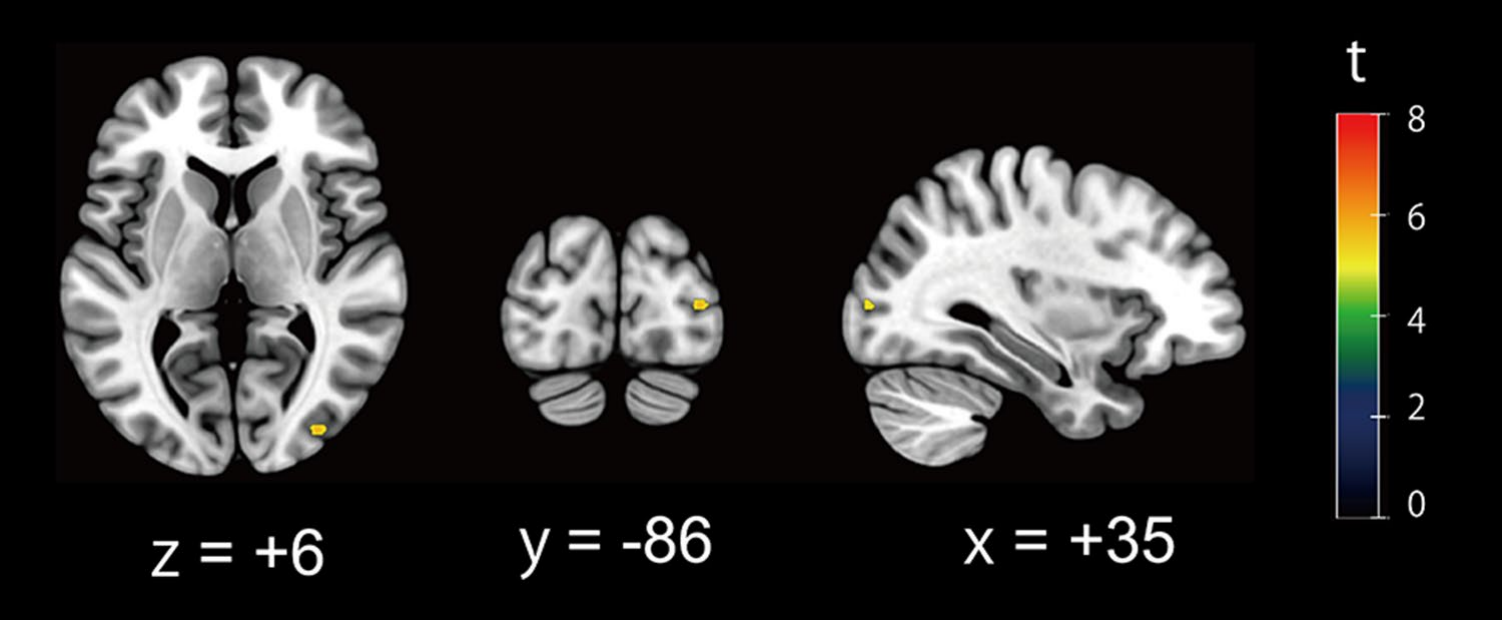
Brain region showing the significant correlation between gray matter (GM) volume and SVBA performance. The significant clusters are superimposed on the axial (left), coronal (center), and sagittal slices (right) of the standardized anatomical image. A color bar represents the *t* value. The GM volume in the right middle occipital gyrus (peak MNI coordinate *x* = 35, *y* =  − 86, *z* = 6; *t* = 5.85; cluster size = 31 voxels) was significantly positively correlated with the TE values in the SVBA task (*p* < 0.05 FWE-corrected at the voxel and cluster levels).

Using the VBM analysis, the cortical region was assessed with respect to errors induced by body tilt (tilt-induced error; TE) in each orientation task. For the SVBA task, we found that the GM volumes in the right middle occipital gyrus (peak MNI coordinate *x* = 35, *y* =  − 86, *z* = 6; *t* = 5.85; cluster size = 31 voxels) were significantly and positively correlated with the TE values (Fig. 3). This result indicates an association between higher GM volumes in this region and larger SVBA bias in the direction of the body tilt. No regions were negatively correlated with TE values, and for the SVV task, no regions were significantly correlated with TE values.

Because the cluster-based thresholding is an arbitrary criterion, we also performed the analysis with the thresholding-free cluster enhancement (TFCE) permutation test^27^. As a result, a significant positive correlation was found between the GM volumes in the bilateral occipito-temporal regions (see Table 1 in detail) and the TE values in the SVBA task (*p* < 0.05 FWE-corrected). Because the bilateral occipito-temporal regions include the peak coordinate of the right middle occipital gyrus found in the cluster-based thresholding, the TFCE result supports the robust association between GM in the region and the ability to estimate the body axis orientation. For TE values in the SVV task or OKS-effect in the SVBA or SVV tasks, no significant clusters were found.

**Table 1:** Brain regions showing significant positive correlation with tilt-dependent error (TE) values in the SVBA task in the thresholding-free cluster enhancement (TFCE) analysis.

Cluster		Peak		MNI		
Region	*k*_E_	Location	*pFWE*	*x*	*y*	*z*
Right occipito-temporal	1200	Middle occipital gyrus	0.004	35	− 86	5
		Middle temporal gyrus	0.008	48	− 72	17
Left occipito-temporal	2733	Inferior occipital gyrus	0.013	− 47	− 78	− 6
		Middle occipital gyrus	0.014	− 51	− 75	− 2
		Middle temporal gyrus	0.018	− 53	− 74	6
		Superior occipital gyrus	0.020	− 18	− 81	29
		Cuneus	0.025	− 14	− 83	30
		Inferior temporal gyrus	0.025	− 54	− 57	− 8
		Calcarine	0.028	− 3	− 90	5

Results are listed at *p* < 0.05 FWE-TFCE-corrected. The extent of each cluster was calculated based on the TFCE *p* value image using the xjview toolbox (http://www.alivelearn.net/xjview).

*k*_E_ cluster size, *pFWE* *p* value FWE-TFCE-corrected, *MNI* Montreal Neurological Institute.

Region of interest (ROI)-based analyses were conducted to examine the involvement of the rTPJ, left parieto-occipital cortex, bilateral posterior parietal, or superior frontal regions in the SVBA or SVV tasks. However, no brain regions showed a significant correlation with the TE values in the SVBA or SVV tasks.

We also explored the cortical involvement in any impact of visual background motion (OKS-effect) on performance in each orientation task. However, no regions were correlated with the OKS-effect in terms of performance on the SVBA or SVV tasks. ROI analysis did not show a significant correlation between the GM volumes in the left PPC and OKS-effect in the SVBA or SVV tasks.

## Discussion

This pre-registered VBM study aimed to identify cortical regions related to perception of the directions of body axis (SVBA) or gravity (SVV). We found a significant correlation between the performance on the SVBA task and GM volume in the right middle occipital gyrus. However, no regions were significantly correlated with performance on the SVV task and visual dependency. These results suggest that the right middle occipital gyrus may play a role in body-centered spatial coding.

The local GM volumes in the right middle occipital gyrus showed significant correlations with the amplitude of TE value in the SVBA task (Fig. 3). Given that the SVBA task is egocentric and does not require participants to consider the body orientation relative to gravity during the task, the above result implies the involvement of this region in egocentric (body-centered) spatial coding. However, the ventrolateral occipito-temporal cortex comprising the middle occipital gyrus, located within the visual ventral stream^28^, subserves visual processing of relative spatial relationships with objects, referred to as allocentric spatial coding^29, 30^. Rather, it has been established that egocentric coding of a visual object underlying goal-directed actions is mediated by the dorsal visual stream^28, 31^. However, when the conscious perception of an external object’s egocentric position or direction is required without planning and controlling goal-directed actions, as in the SVBA task, the ventral visual stream may also be involved in egocentric spatial coding^32^. Several studies have reported the activation in areas of the ventrolateral occipito-temporal cortex, such as the occipital^9, 10, 29, 33^ and lateral temporal cortices^10, 33^, during an egocentric judgment task. Furthermore, a recent meta-analysis using activation likelihood estimation (ALE) has shown the specific involvement of the right middle occipital gyrus to egocentric spatial judgment but not to allocentric judgment^34^. The present results support this finding and suggest a specific contribution of the right middle occipital gyrus to egocentric spatial coding. However, considering the TFCE-based results showing the significant clusters in the bilateral occipito-temporal regions (Table 1), it is possible that the bilateral cortical region comprising the ventral pathway may be involved in the perception of the body axis orientation.

Previous fMRI studies have shown that other cortical regions, such as the bilateral posterior parietal or frontal cortices, are also activated during body-centered judgment of a visual target^10–12^. However, the present VBM analysis revealed no significant correlations between the GM volumes in these cortical regions and SVBA performance. The reason for the discrepancy between our results and those of previous studies is unclear. One possible explanation might be the difference in posture during the task. Although the participants performed the body-centered judgment task in the supine position in previous fMRI studies, the participants’ bodies were tilted in this study. Lateral body tilt alters the pattern of afferent sensory inputs, such as ocular torsion biasing the opposite direction of head tilt^35^, asymmetrical distribution of the tactile (pressure) stimulus^36^, and a decrease in otolith (utriculus) sensitivity^37^. Therefore, when the body is tilted, the CNS needs to recalibrate the internal representation of egocentric space in response to the tilt-dependent change of sensory information for accurate estimates of the body axis orientation. We speculate that although the parieto-frontal network is responsible for egocentric spatial coding, it may not strongly engage in the recalibration process of egocentric spatial representations according to body orientation in space. Alternatively, the lack of a significant correlation might be due to a methodological limitation of VBM, which generally assumes a linear relationship between behavioral performance and local GM volumes. Accordingly, if the relationship is non-linear, the sensitivity of the method may be reduced.

We observed that greater GM volumes in the right middle occipital gyrus were correlated with larger SVBA biases (i.e., worse performance). The direction of this relationship appears contradictory to the concept that increased GM is associated with better performance, supported by many VBM studies on sensorimotor (e.g.,^38^), perceptual (e.g.,^39^), and cognitive performances (e.g.,^40^). One potential reason for this difference might be synaptic pruning during cortical maturation^17^. It is known that in the development stage, the brain removes weak synapses while stronger connections are strengthened, leading to a reduction in the cortical volume and enhancement of the efficiency of neural transmission^41^. Some studies have reported an inverse correlation between individual performance and the local GM volume/density and interpreted these findings based on neural pruning^42–44^.

In contrast to previous studies^13–15, 26^, the present study did not find significant correlations between any GM volumes and SVV performance (TE value) or visual dependency (OKS-effect). In addition to the abovementioned limitation of VBM analysis, the following explanations are feasible. First, the number of participants (*n* = 50) may have been insufficient. The involvement of these regions with SVV performance or visual dependence may be relatively weak, and thus more participants may be needed to detect significant clusters. Second, in the present study, the body tilt angles (10°) and the visual angle of the OKS presentation (24.8°) were relatively small. Neither of these factors strongly affect the estimated gravitational direction^45, 46^, perhaps reducing the likelihood of detecting inter-individual differences in the SVV performance or visual dependency. Further studies using larger body angles and wider visual angles are needed to examine this possibility. Third, vestibular function may contribute to performance on visual vertical estimates and visual dependency. A previous study demonstrated an association within individuals between ability to perceive gravity-centered space and otolith function^47^. Other studies have shown that vestibular function is an important determinant of visual dependency when estimating the directions of body axis or gravity^48, 49^. Thus, we speculate that differences in SVV performance or visual dependency across participants might be derived from individual otolith function rather than from multisensory integration in the brain.

In conclusion, the present VBM study shows a significant correlation between GM volumes in the right middle occipital gyrus and ability to estimate the body axis orientation. This finding provides evidence on the neuroanatomical substrates of body-centered spatial coding during body tilt. Further intervention studies using brain stimulation such as TMS are needed to assess a causal link between the implicated brain region and perception of body axis orientation.

## Methods

### Participants

Fifty right-handed healthy participants (25 men and 25 women, aged 20–46 years) were recruited. All participants reported having normal vision and no cognitive, neurological, or sensorimotor disorders. Prior to the experiments, all participants provided written informed consent to participate in this study and completed a questionnaire about their experiences with respect to car driving and sporting activities. The sample size was determined based on previous VBM studies^39, 42^ on the correlation between individual perceptual ability and local GM volumes. The present study was approved by the Ethical Committee of Hamamatsu University School of Medicine and was conducted in accordance with the Declaration of Helsinki. The study protocol was pre-registered in the University Hospital Medical Information Network (registration number: UMIN000036806).

### Measurement of behavioral performance

#### Experimental setup

Participants sat on a tilting chair (SP-PS100-Z, Pair support, Japan), which could be rotated in the roll plane, and their head, trunk, and legs were firmly secured to the seat with bands and a seat belt. The maximum velocity and initial acceleration of the tilting chair were set at 0.69°/s and 1.06°/s^2^, respectively, which is below the threshold of the semicircular canal stimulation^50^. A display (width: 12.6 cm, height: 17.1 cm) was placed at a viewing distance of 25 cm in the line of sight. To prevent the participants from obtaining any visual cues (e.g., the edge of the display) other than the visual line, a black cylinder (26 cm in diameter), one side of which was covered by a black board with a hole (11.0 cm in diameter), was inserted between the participant’s head and the display. During the experiment, participants were provided with white noise via earphones to mask auditory spatial cues from the surrounding environment.

#### Spatial orientation task

Participants performed either the SVBA task or the SVV task 10 times consecutively with each visual background in upright or roll-tilted (LSD or RSD) positions.

**Figure 4:**
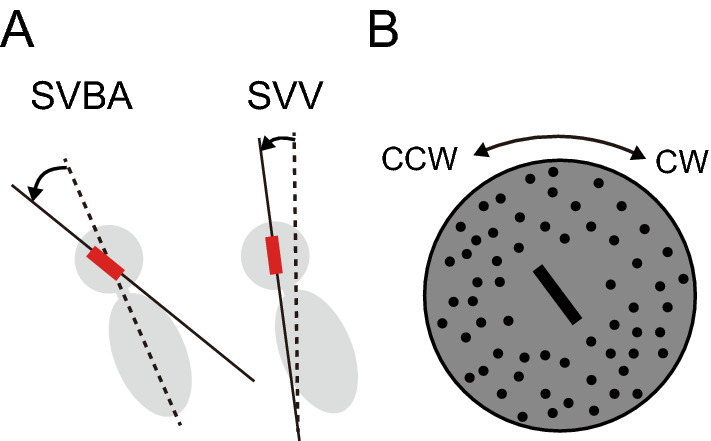
Schematic illustration of each spatial orientation task and visual stimuli. (**A**) Participants were asked to align the visual line (denoted as red lines) along the perceived direction of the body longitudinal axis (SVBA task) or gravitational vertical (SVV task). Adjustment errors in each task (denoted as arrows) were calculated as the angular bias of the subjective directions of body longitudinal axis or vertical (solid lines) from the objective directions (dotted lines). (**B**) Illustration of the visual stimuli on the display as seen by the participants in the optokinetic stimulation (OKS) conditions. Many dots were randomly positioned around the visual line, and they rotated uniformly clockwise (CW) or counterclockwise (CCW) around the center of the display during the SVBA or SVV tasks.

For the SVBA task, participants adjusted a visual line (4.6 cm in length) presented at the center of the display along the perceived direction of the body longitudinal axis using a controller. For the SVV task, they adjusted the line along the perceived direction of gravity (Fig. 4A). Note that the SVBA task is egocentric, not requiring participants to consider their body tilt angle relative to gravity, unlike the SVV tasks. For both tasks, the initial angle of the line was randomly set at ± 45°, ± 60°, or 90° with respect to the longitudinal axis of the participant’s body. After repeating 10 times, participants were tilted back to the upright position.

One of the three visual background conditions (CW-, CCW-, or No-OKS) was presented during each spatial orientation task. In the CW- and CCW-OKS conditions, random dots were rotated around the center of the display clockwise or counterclockwise from the participants’ perspective at 30°/s (Fig. 4B). The OKS was presented within a viewing angle of 24.8°, excluding an area with a radius of 4.6 cm from the center. The diameter of each dot and the density of dots were 2.5 mm and 8.7 dots/cm^2^, respectively. In the No-OKS condition, no visual dots were presented during the task.

Each participant performed each orientation task (SVBA or SVV) 10 times in each of the nine conditions, a combination of three visual background conditions (CW-, CCW-, or No-OKS) and three body tilt conditions (0°, LSD or RSD 10°), that is, 90 times for each task. The SVBA and SVV tasks were performed in separate blocks with nine conditions. The order of visual background and body tilt conditions within each block was randomized for all participants.

#### Performance evaluation

We quantified the individual performance in terms of accuracy in each orientation task. An adjustment error was computed for each trial as the angular difference between the adjusted line and the objective direction (body longitudinal axis for the SVBA task, gravitational vertical for the SVV task). The mean adjustment error was calculated for each of the 10 trials in each visual background and body tilt condition. Using only the data of the No-OKS condition, the TE value was calculated for each orientation task by computing the absolute difference in the adjustment error between the 0° position and the LSD 10° or RSD 10° positions and calculating the mean. A lower TE value indicates that the performance on each task was less affected by the body tilt.

Additionally, we quantified the effect of visual background motion on each orientation task for each participant. The OKS-effect was computed by subtracting SVBA or SVV errors in the No-OKS condition (i.e., baseline) from those in CW- or CCW-OKS conditions at each body tilt position. Positive and negative values of the data were replaced only for CCW-OKS to redefine the OKS-effect as the bias toward the rotation direction of OKS. Finally, the mean OKS-effect was calculated for all OKS (CW- and CCW-OKS) and all body tilt conditions (0°, LSD10°, or RSD10°), which were used as representative values for each participant.

### VBM analysis

#### Magnetic resonance imaging acquisition

T1-weighted magnetic resonance images were acquired using a 3.0 T MR imaging scanner (Discovery MR750 3.0 T, GE Healthcare Japan, Japan) with a 12-channel head component of the head-neck-spine array coil. The following parameters were applied for MRI: repetition time = 7.2 ms, echo time = 2.1 ms, flip angle = 15°, field of view = 256 mm × 256 mm, voxel size = 1 mm × 1 mm × 1 mm, and matrix = 256 × 256.

#### Preprocessing of MR images

To evaluate local GM volumes, VBM analysis was conducted using the Statistical Parametric Mapping (SPM) 12 software (http://www.fil.ion.ucl.ac.uk/spm) in a MATLAB version 9.8.0 (R2020a) environment. After visually checking the artifacts on the T1-weighted MR image of each participant, the following conventional data preprocessing steps were taken: (1) setting the image origin on the anterior commissure, (2) correction of the intensity inhomogeneity due to the bias field, (3) segmentation of different tissue classes, (4) linear (affine) and nonlinear spatial normalization to the Montreal Neurological Institute (MNI) stereotaxic standard space using the Diffeomorphic Anatomical Registration using Exponentiated Lie Algebra (DARTEL) template, and (5) modulation of the different tissue segments using nonlinear normalization parameters to correct for differences in brain size between participants. The DARTEL template was constructed from 555 healthy control participants in the IXI-database (http://brain-development.org/). The voxel size was resampled to 1.5 × 1.5 × 1.5 mm. Finally, normalized GM segments were smoothed with an 8-mm full-width half-maximum Gaussian kernel. After completing these preprocessing steps for all participants, statistical analyses were performed.

#### Statistical analysis

To identify the cortical region in which the GM volume positively or negatively correlated with each performance (TE or OKS-effect) in the SVBA or SVV tasks, multiple regression analyses were conducted. In each analysis, participants’ sex, age, days between the date of MRI and behavioral assessment (1–495 days), and total brain volume calculated as the sum of GM and WM volume were included as nuisance covariates. Because experiences with daily driving and sports influence visuospatial perceptual ability^51^ and GM volume^52^, these factors may cause a pseudo-correlation between each performance and the local GM volume. Therefore, the number of years of daily driving (0–27 years) and sports experience (0–16 years) were also used as nuisance covariates. We applied an absolute threshold mask of 0.2. The statistical threshold was set at *p* < 0.05 corrected for multiple comparison (family-wise error; FWE) at both voxel and cluster levels. The Automated Anatomical Labeling (AAL) 3 toolbox (https://www.gin.cnrs.fr/en/tools/aal/) for SPM12 was used to label the significant cluster.

According to one reviewer’s suggestion, the TFCE was also performed as a supplemental analysis^27^. All TFCE-based analyses were conducted with 5000 permutations and a significance level of *p* < 0.05 (FWE-corrected) using CAT12 toolbox version 12.8 (http://dbm.neuro.uni-jena.de/cat/) in SPM12.

We also performed ROI-based analyses in accordance with previous studies (e.g.,^13^). The target ROIs were defined as 8-mm spheres. The center of each ROI was determined based on previous studies as follows: MNI coordinates *x* = 61, *y* =  − 39, *z* = 22 for rTPJ^15^, *x* =  − 23, *y* =  − 89, *z* = 31 for the left parieto-occipital cortex^13^, *x* = 28, *y* = 2, *z* = 52 and *x* =  − 28, *y* =  − 7, *z* = 56 for the bilateral superior frontal cortex, and *x* = 16, *y* =  − 65, *z* = 58 and *x* =  − 24, *y* =  − 65, *z* = 53 for the bilateral posterior parietal cortex^10^. The center of the ROI for the left PPC was determined at *x* =  − 40, *y* =  − 76, *z* = 47 by calculating the MNI coordinates corresponding to the P3 position (international 10–20 system for EEG electrode placement) with reference to a previous study^53^. The MarsBaR toolbox (http://marsbar.sourceforge.net/) was used to create the ROI image. Small volume correction was conducted for each ROI region to assess the correlations between the GM volume and TE or OKS-effect values in the SVBA or SVV tasks.

## Data Availability

The datasets analyzed in the present study are available from the corresponding author upon reasonable request.
